# The Depleting Impact of Helping Behavior on Career Satisfaction: The Buffering Role of Strengths Use

**DOI:** 10.3390/ijerph20010161

**Published:** 2022-12-22

**Authors:** Zhigang Li, Zhenduo Zhang, Qian Li, Junwei Zheng, Huan Xiao

**Affiliations:** 1 School of Economics and Management, Beijing Polytechnic, Beijing 100176, China; 2 School of Economics and Management, Dalian University of Technology, Dalian 116024, China; 3 Faculty of Civil Engineering and Mechanics, Kunming University of Science and Technology, Kunming 650500, China; 4 School of Management, Harbin Institute of Technology, Harbin 150001, China

**Keywords:** helping behavior, perceived task demands, job strain, strengths use, career satisfaction

## Abstract

Recent studies have explored the dark side of helping behavior from an actor-centric perspective. Consistent with this stream of research, this study linked helping behavior to career satisfaction. In this study, we adopted perceived task demands and job strain as two sequential mediators to elaborate the underlying depletion path through which helping behavior undermines career satisfaction. We collected data using a two-wave questionnaire completed by 203 full-time workers in China. By applying path analysis using R software, the results revealed the following: (1) helping behavior undermines career satisfaction by enhancing perceived task demands and job strain; (2) the use of strengths buffers the relationship between perceived task demands and job strain; and (3) the indirect depleting impact of helping behavior on career satisfaction only emerges when the use of strengths is low. This highlights important implications for practitioners to leverage helping behavior in their management practices.

## 1. Introduction

Researchers and practitioners consider employee helping behavior to be an effective tool for enhancing team cohesion and performance [1]. Helping behavior is defined as voluntary assistance given to coworkers to accomplish goals or prevent problems [2]. Mainstream research has explored the antecedents of helping behavior, such as leadership style, organizational climate, and specific employee traits [3,4,5]. Recently, however, studies have begun to explore the outcomes of helping behavior from an actor-centric perspective, especially the dark side of helping behavior. For instance, helping behavior may impede helpers’ work progress, take up extra work time, and increase ego depletion [6,7,8].

Past studies have provided useful insights into actor-centric outcomes of helping behavior, focusing on the impact of helping behavior on employees’ current job performance and job satisfaction; however, little attention has been paid to how it affects employees’ long-term growth and development through measurements such as career satisfaction. Career satisfaction refers to a sense of pleasure for employees in their careers and a positive belief that employees can expect to succeed in their career [9]. Although both career satisfaction and job satisfaction focus on employees’ psychological satisfaction, there are essential differences between the two concepts. Specifically, job satisfaction mainly focuses on the employees’ satisfaction with work itself (such as work environment, work pressure, interpersonal relationships in the workplace, etc.), while career satisfaction focuses more on the employees’ satisfaction with their progress, prospects, and significance at work [10]. Career satisfaction determines employees’ intention to stay in the organization and the extent to which they engage in their work, which is a key factor promoting the sustainable development of the organization and creating the best organization for the employer [11]. Therefore, exploring the association between helping behavior and career satisfaction can provide insight into the long-term outcomes of helping behavior at work, which can be leveraged for improved employee management.

Work time is limited, and employees need to complete assigned tasks within specific periods [12]. Helping behavior requires time to help coworkers address work difficulties [7,12]. As a result, employees are confronted with increased task demands and need to work extra hours to complete assigned tasks [13,14]. High task demands occur when employees perceive there is a great amount of work to be completed under time pressure, requiring them to work faster and harder [15]. Job strain is a psychological response to perceived task demands. Previous studies have explored the positive relationship between perceived task demands and job strain. Job strain is a subjective reaction to stressful work conditions or events evoking an unpleasant emotional experience [16,17]. This can stifle career satisfaction [18]. Therefore, in this study, we adopted perceived task demands and job strain as two sequential mediators to reveal the underlying depletion mechanism by which helping behavior impedes career satisfaction.

Prior studies also identified a positive relationship between perceived task demands and enhanced job satisfaction [19]. The job demands–resource (JD-R) model proposes that the impact of job demands on employees’ psychological and behavioral outcomes depends on the resources they possess [20,21]. The use of strengths, or strengths use, represents the employees’ initiative in applying their strengths at work [22]. Strengths use is recognized as a job resource that facilitates an employee’s ability to cope with task demands, enhancing their positive psychological state and promoting career satisfaction [23,24]. Therefore, we adopted strengths use as a moderator to explore when perceived task demands caused by helping behavior lead to job strain and decreased career satisfaction.

Previous studies identified that helping behavior can be transformed into job demands that impede work progress and lead to ego depletion [5,7]. This study was developed using the framework of the JD-R model. Perceived task demands and job strain were adopted as two sequential mediators, and strengths use served as a moderator to assess how and when helping behavior stifles career satisfaction. A two-wave survey was used to collect data to test these relationships.

The study makes two key contributions to the helping behavior literature and the JD-R model. First, this study uncovers the depletion mechanism through which helping behavior impacts career satisfaction. Second, this study describes the boundary condition under which helping behavior impedes career satisfaction through perceived task demands and job strain by exploring the moderating role of strengths use.

## 2. Hypothesis Development

### 2.1. Helping Behavior and Perceived Task Demands

Time is a limited and valuable resource at work. Workdays are segmented into finite episodes associated with specific work goals [25]. Employees strive to make progress toward work goals [26]. However, helping behavior is a time-dependent behavior by which limited time resources are invested to help coworkers address relevant difficulties. Helping behavior is a process that transfers time resources from helpers to recipients [27]. For helpers, there is a trade-off between the time assigned for task performance and the time needed for helping behavior [28]. Helpers have limited time to complete their core tasks in order to make up for their slowed work progress, leading to an increase in perceived task demands.

Moreover, helping behavior usually occurs in response to a request for aid; such requests tend to disrupt ongoing performance [29]. Prior studies have shown that cognitively shifting attention from core tasks to requests for help further impede work-related progress [12,30]. Helpers may have to actively turn off their thoughts associated with their tasks to channel their energy toward responding to the request for help [5]. This means that helping behavior may also decrease the helpers’ cognitive ability to complete tasks and increase their stress level when they are required to complete assigned tasks within a limited amount of time. This circumstance leads to the following hypothesis:

**Hypothesis 1 (H1)**: *Helping behavior is positively associated with perceived task demands.*

### 2.2. Perceived Task Demands and Job Strain

Job strain has been examined as an important work-related consequence of hindrance demands [31,32]. Job demands are specific aspects of a job that consistently deplete personal resources [21]. Job strain is determined by the interaction between an employee’s job resources and the work environment. When the job demands are perceived to exceed the employee’s coping abilities, job strain can occur.

High task demands reflect the amount of work to be done and the need for employees to work harder and faster under time pressure [15]. Previous studies have found that individuals tend to recall negative work events as a maladaptive response to high task demands [33]. Employees experiencing high task demands may not easily disengage from work-related matters due to their high-level engagement in the task to meet job requirements [34]. Given this tendency for rumination, prior studies have explored the positive relationship between perceived task demands and several negative affective reactions, such as depression and anxiety. As discussed above, job strain reflects an unpleasant emotional reaction induced by stressful events. Taking all of this together, we hypothesized the following:

**Hypothesis 2 (H2)**: *Perceived task demands are positively associated with job strain.*

### 2.3. Job Strain and Career Satisfaction

Career satisfaction represents subjective career success, indicating an employee’s experience and evaluation of personally meaningful career outcomes [35,36]. Job strain is a key determinant of career satisfaction. For example, Grimland et al. (2012) addressed individual reactions to stressful events and the mechanisms used to address them.

The JD-R model proposes that job strain consistently depletes and threatens an individuals’ job resources [21]. As a result, job strain leads to more serious negative health outcomes, such as psychosomatic disease, emotional exhaustion, and depression [21]. Responses to stressors (e.g., perceived task demands) may reflect the current unsatisfactory situation, indicating that a change is needed to adjust the status quo [17]. Job strain is an unpleasant emotional reaction to a mismatch between stressful work conditions and personal resources, leading to a decreased subjective evaluation of the meaning and value of the current job [20]. This leads to the following hypothesis:

**Hypothesis 3 (H3)**: *Job strain is negatively associated with career satisfaction.*

As noted above, helping behavior describes when employees invest limited work time to help coworkers address work-related difficulties. As a result, helpers usually face increased perceived task demands. When the task demands exceed the helpers’ job resources, they will have a sense of job strain. This job strain represents their dissatisfaction with the current work conditions, leading to stifled career satisfaction. This leads to the following hypothesis:

**Hypothesis 4 (H4)**: *Perceived task demands and job strain sequentially mediate the relationship between helping behavior and career satisfaction.*

### 2.4. Moderating Role of Strengths Use

Previously, perceived task demand was defined as a hindrance demand associated with role stress, increased work fatigue, and increased work–family conflict [15]. However, studies have also addressed the beneficial aspects of perceived task demand. For example, Schyns and Croon (2006) explored the positive relationship between perceived task demand and job satisfaction [19]. This paradoxical outcome provided by the JD-R model may be due to a lack of inclusion of employee-held resources. To clarify the boundary conditions under which perceived task demands induced by helping behaviors lead to job strain, in this study, we adopted strengths use as a moderator that facilitates a helper’s ability to cope with task demands.

Strengths use reflects the extent to which individuals are familiar with and take advantage of their strengths at work [22]. Prior studies have found that strengths use enhances a sense of thriving at work, positive affective experience, and self-efficacy. These facilitate individuals’ ability to cope with job demands, achieve work goals, and maintain a positive psychological state [37,38]. When individuals face increased task demands, those with high strengths use take advantage of their strengths to develop innovative and effective strategies to complete assigned tasks and catch up on slowed work progress [39]. As such, perceived task demands will not result in job strain. In contrast, those with low strengths use are more likely to be trapped in assigned tasks, use more time to catch up on work progress, and tend to be over-engaged in their jobs, leading to negative cognitive and emotional reactions to task demands. Taking all of this together, this study hypothesizes the following:

**Hypothesis 5 (H5)**: *Strengths use moderates the relationship between perceived task demands and job strain, and a positive association between perceived task demands and job strain emerges when strengths use is low.*

As discussed above, perceived task demands and job strain may sequentially mediate the relationship between helping behavior and career satisfaction. However, this depletion path depends on strengths use. Those with high strengths use can complete assigned tasks and catch up on delayed work progress by using their strengths at work. In contrast, those with low strengths use tend to complete their tasks routinely, increasing job strain. This study identifies strengths use as a resource that assists helpers in coping with increased task demands, consistent with previous JD-R model research. It is assumed that the depleting impact of helping behavior on career satisfaction emerges in the condition of low strengths use. Therefore, we hypothesize the following:

**Hypothesis 6 (H6)**: *Strengths use moderates the indirect depletion relationship between helping behavior and career satisfaction, and this indirect relationship is significant when strengths use is low.*

**Figure 1:**
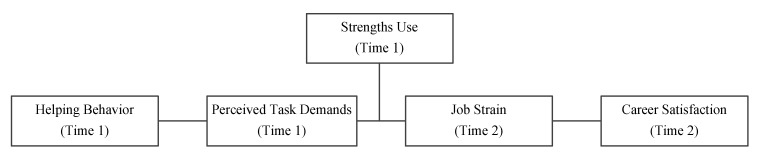
Conceptual model.

The conceptual model can be seen in Figure 1.

## 3. Method

### 3.1. Participants and Procedure

Data were collected from employees working for a construction company in Beijing, China, which has over 1000 employees. The research began in October 2020 and ended in December 2020. We sent 250 questionnaires to human resource managers and asked for their help in sending the questionnaires to employees. Before we started the data collection, we trained the 2 human resource managers on the 2-step data collection procedure and precautions. To control for common methodological biases, survey data were collected at 2 time points one month apart. At time 1, participants were asked to report their helping behavior, perceived task demands, and strengths use. At time 2, one month after time 1, participants rated their job strain and career satisfaction. At both time points, participants were asked to report their demographic information and code name (their initials), which were used for survey matching.

The distribution and recovery of the questionnaires was performed by the 2 human resource managers. One of the human resource managers sent emails to the employees of the company to inquire about their intention to participate in the survey, and over 400 employees were willing to take part. Subsequently, 250 samples were chosen randomly from various departments by the human resource managers. The questionnaires and informed consent forms were sent to the human resource managers as hard copies, and they were responsible for resealing, packaging, and returning the completed questionnaires and informed consent forms in a sealed envelope to the research team. We also recorded a video explaining informed consent for the human resource managers to play for the participants before the data collection. The participants were notified that when they completed the first wave of the questionnaire, they would receive RMB 15 (≈USD 2.09). When they completed both parts of the questionnaire, they received RMB 50 (≈USD 6.95). The high reward for completing both parts of the questionnaire was adopted to ensure an effective response rate. 

In total, 227 employees returned the questionnaire at time interval 1, and 203 employees responded to the second survey at time interval 2. The resulting effective response rate was 81.2%. Among the participants, 48.8% were male and 51.2% were female at birth; 18.7% had a junior college certificate or below, 63.1% had a bachelor’s degree, and 18.2% had a master’s degree or above; 39.4% were married and 61.6% were unmarried; and 83.3% were frontline white-collar workers and 16.7% were white-collar team leaders. All participants worked in the construction industry. The average age of the participants was 32.27 years (SD = 6.34) and their average tenure at the organization was 5.78 years (SD = 5.29).

### 3.2. Measures

All instruments used in this study are published in academic journals and available for public use in academic studies. They were originally developed in English. As such, a translation-back translation procedure was adopted to ensure translation accuracy [40]. Without special statements, a 5-point Likert scale was used, generally ranging from 1 = strongly disagree to 5 = strongly agree. The items used to measure each construct and the pre-validated scales referred to are presented in Supplementary Materials.

Helping behavior. Three items developed by Yue et al. [8] were used to measure helping behavior. A sample item is, “I help my colleagues when it is clear their workload is too high”. A 5-point Likert scale was used to measure how frequently employees engaged in helping behavior last month, with 1 indicating never and 5 indicating always. The internal reliability of this scale was 0.79.

Perceived task demands. Perceived task demands were measured using 4 items developed by Williams and Alliger [15]. A sample item is, “I needed to work hard on my work”. The scale yielded a Cronbach’s alpha of 0.71.

Job strain. Two items developed by Motowidlo et al. [41] were used in this study to measure job strain; these items were also used by Schmitt et al. [17] to verify item reliability. The 2 items are, “I feel a lot of pressure at work” and “I often feel too tense due to my work”. The Cronbach’s alpha of this scale was 0.86.

Career satisfaction. Career satisfaction was measured using 5 items developed by Eby et al. [42]. A sample item is, “I am satisfied with the success I have achieved in my career”. This scale yielded a Cronbach’s alpha of 0.81.

Strengths use. We used 4 items developed by van Woerkom et al. to measure strengths use [22]. A sample item is, “I have benefited my work from my strengths”. The Cronbach’s alpha of this scale was 0.88.

Control variables. Gender, age, and education were used as control variables since all are commonly included factors that are likely to be related to career satisfaction. Existing research has identified gender, age, and education as key demographic variables that influence career satisfaction [35,36].

### 3.3. Ethical/IRB Review

Participants were informed that the collected data would be used only for research purposes and their personal data would not be disclosed to protect their privacy. They were also informed about the research purpose and process. They were required to sign an informed consent form at the beginning of the first wave of the survey to ensure that they understood they had the right to quit the research at any time. This study was guided by the principles of the Declaration of Helsinki and approved by the Ethical Committee of Kunming University of Science and Technology (2021-KYLX02-02).

## 4. Results

### 4.1. Confirmatory Factor Analysis and Descriptive Analysis

Before performing descriptive analysis and testing the hypotheses, we performed confirmatory factor analysis to examine the discriminant validity of the scales operationalized in this study using R (V4.1.1) and the Lavaan package in R (V0.7.2). Table 1 shows the results. The proposed five-factor model had the best fit ($\chi^{2}\;$= 236.52, df = 124, SRMR = 0.07, CFI = 0.92, TLI = 0.91, RMSEA = 0.07) compared to other models (Δ$\chi^{2}\;$≥ 100), indicating the scales had an acceptable level of validity.

Table 2 lists the results of the descriptive analysis, including means, standard deviations, and correlations of all variables using R (V4.1.1) and the Bruce R package (V0.7.2) (R Foundation: Vienna, Austria).

### 4.2. Hypothesis Tests

To test the conceptual model, a path analysis was conducted using R (V4.1.1) and the Lavaan package (0.6–12). Path analysis is a multivariate methodology for empirically examining sets of relationships represented in the form of linear causal models [43]. Path analysis has been used to examine complex conceptual models statistically, and examples of path analysis applications can be found in many occupational psychological studies [44,45]. 

**Figure 2:**
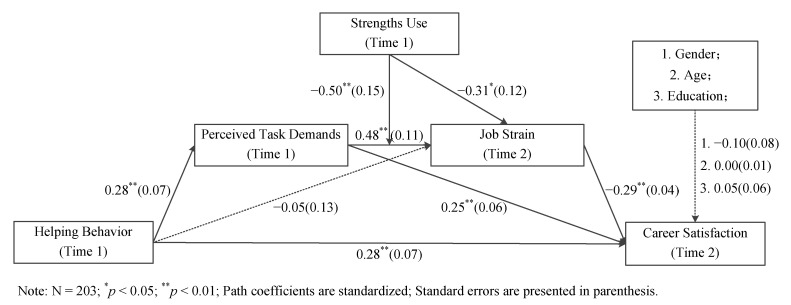
Results of path analysis.

Figure 2 shows the results. Helping behavior was positively correlated with perceived task demands (β = 0.28, SE = 0.07, *p* < 0.01), supporting Hypothesis 1 (H1). Perceived task demands were positively correlated with job strain (β = 0.48, SE = 0.11, *p* < 0.01), supporting Hypothesis 2 (H2). Job strain was negatively associated with career satisfaction (β = −0.29, SE = 0.04, *p* < 0.01), supporting Hypothesis 3.

To examine the sequential mediating roles of perceived task demands and job strain in the relationship between helping behavior and career satisfaction, bootstrapping analysis was performed, and the results are shown in Table 3. The multiple mediation effect was significant (Effect = −0.04, SE = 0.01, 95% CI = [−0.07, −0.01]), supporting Hypothesis 4.

**Figure 3:**
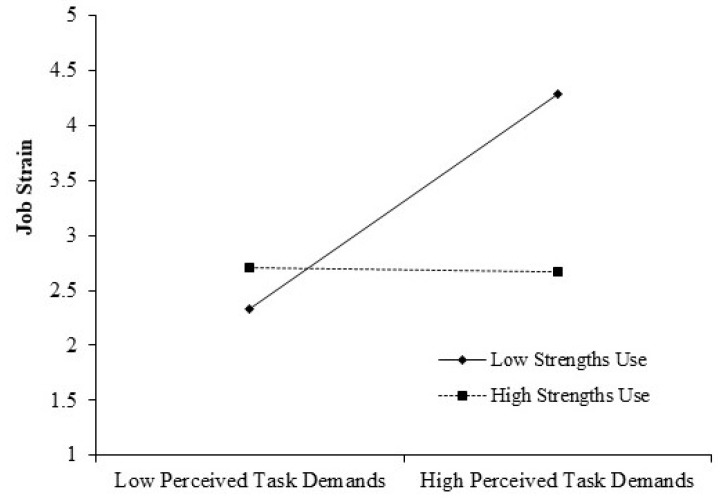
Results of the moderating effect of psychological entitlement.

Concerning the moderating role of strengths use, the interactive item of perceived task demands with strengths use was positively associated with job strain (β = 0.48, SE = 0.11, *p* < 0.01). We also conducted a simple slope test, and the results are shown in Table 3 and Figure 3. When strengths use was low, the relationship between perceived task demands and job strain was significant (Effect = 0.79, SE = 0.14, 95% CI = [0.51, 1.07]). When strengths use was high, this relationship was not significant (Effect = 0.18, SE = 0.14, 95% CI = [−0.10, 0.46]). The difference between these two slopes was significant (Effect = −0.61, SE = 0.18, 95% CI = [−0.97, −0.25]), indicating the significant moderating role of strengths use. This supports Hypothesis 5 (H5).

We also examined the moderated multiple mediation model using a bootstrapping analysis. The results in Table 3 indicate that the sequential mediating effect of perceived task demands and job strain was significant when strengths use was low (Effect = −0.06, SE = 0.02, 95% CI = [−0.11, −0.03]). In contrast, when strengths use was high, the indirect relationship was not significant (Effect = −0.01, SE = 0.01, 95% CI = [−0.04, 0.01]). The difference between these two effects was significant (Effect = −0.05, SE = 0.02, 95% CI = [0.02, 0.10]). These results support Hypothesis 6 (H6).

## 5. Discussion

Based on the JD-R model, this study explored how and when helping behavior undermines career satisfaction from an actor-centric perspective. We adopted perceived task demands and job strain as two sequential mediators, and strengths use served as a moderator. The results of our data analysis support all the hypotheses proposed in the present study. First, our results found that helping behavior impedes career satisfaction by enhancing perceived task demands and job strain. Employees spend their limited working time helping others. Accordingly, they must reduce time spent on completing tasks within their responsibilities, which will make employees perceive higher task demands. High job strain may consistently deplete and threaten employees’ job resources, leading to job strain [21], and previous studies have identified the negative relationship between job strain and career satisfaction [18]. Second, our results proved employees with high strengths use are less likely to increase their job strain after perceiving task demands. Similarly, our results indicated the indirect depletion relationship between helping behavior and career satisfaction only emerges when strengths use is low. Strengths use is a resource that assists helpers in coping with increased task demands. Employees with high strengths use can maintain positive psychological states [37] and formulate effective strategies to catch up on task progress when they face increased task demands [39]. As such, helping will not decrease career satisfaction. The specific theoretical and practical implications are further discussed below.

### 5.1. Theoretical Implications

This study makes two contributions to the current research. First, this study reveals the depletion mechanism through which helping behavior stifles career satisfaction. Few studies have explored the association between helping behavior and career satisfaction. Career satisfaction is a determinant driving individual and organizational outcomes, such as retention, happiness, and capability development [46,47,48]. It also reflects an ideal state in which employees are motivated to achieve in a boundaryless career [48]. Consistent with previous studies on the depleting effect of helping behavior from an actor-centric perspective [6,7,8], this study found that helping behavior enhances perceived task demands and increases job strain, thus impeding career satisfaction. Therefore, our findings extend insights on the outcomes of helping behavior into the field of “career”, providing a useful supplement to previous studies, and also providing a valuable theoretical framework for an in-depth understanding of the mechanism of helping behavior on employee career satisfaction.

As a second contribution to the field, this study found that the indirect depletion relationship between helping behavior and career satisfaction through perceived task demands and job strain depends on the helpers’ use of strengths; this depletion impact only emerged when strengths use was low. This may be because helping behavior could be transformed into job demands for helping actors, leading to unfavorable outcomes [5]. At this time, a lack of strengths use makes it difficult for helpers to complete the assigned tasks effectively [39]. The routine method used to finish tasks propels helpers to invest extra time to catch up on impeded work progress [7]. This increases the likelihood that helpers will experience job strain [15]. These results are in line with the existing studies, which provided indirect evidence of the buffer effect of strengths use. For instance, Bakker and van Woerkom (2018) stated strengths use is recognized as a job resource that facilitates an employee’s ability to cope with task demands [39]. And The JD-R model addresses the buffering role of job resources in coping with job demands. Thus, using the JD-R model framework, this study identified strengths use as a job resource, which may undermine the depleting impact of helping behavior on career satisfaction. This may contribute to a better understanding of helping behavior and the JD-R model.

### 5.2. Practical Implications

This research also has implications for practitioners. First, managers should consider the costs of helping behavior. Our results suggest that helping behavior increases perceived task demands and job strain, thereby decreasing career satisfaction. Managers need understand, first, that overemphasizing helping others may cause the helpers to feel strained and impede their career satisfaction. In order to reduce the negative impact of helping behavior for the helpers, the manager can appropriately reduce the task requirements for them, so as to reduce their resource depletion.

Second, this study suggests that strengths use may buffer the indirect negative relationship between helping behavior and career satisfaction. This negative relationship only emerges for employees with low strengths use. Based on this result, we have two suggestions for managers. On the one hand, organizations could add a measurement of strengths use in the recruitment process and select potential employees with high strengths use. On the other hand, for recruited employees with low strengths use, organizations could provide interventions to enhance their strengths use. Managers could offer a strengths-based training program or an intervention based on the managerial practices in the organization, which could help employees accurately identify their explicit strengths and tap into their implicit strengths, thus increasing the level of strengths use in employees. This could ultimately decrease the depleting impact of helping behavior on career satisfaction.

### 5.3. Limitation and Future Research

Like all studies, this one had some limitations, which highlight opportunities for future research. First, we cannot rule out common method variance (CMV) [49]. We adopted a two-wave survey method to decrease CMV, and the CFA results indicate that CMV was not a serious problem in this study. However, potential bias due to CMV cannot be fully eliminated. Future research should adopt a multi-source and multi-wave research design to avoid CMV.

Second, we were not able to establish a firm causal relationship between helping behavior and career satisfaction. This is because we did not manipulate the dependent variable of helping behavior; as such, we cannot infer its causal impact on perceived task demands, job strain, and career satisfaction. Future studies could adopt a field experimental design or cross-lagged analysis to explore the causal relationship between helping behavior and career satisfaction.

Third, the beneficial impacts of helping behavior on career satisfaction should be further explored. The direct relationship between helping behavior and career satisfaction was significant (B = 0.28, SE = 0.07, *p* < 0.01). These results indicate the presence of a beneficial path through which helping behavior enhances career satisfaction. Future research could explore the indirect beneficial impact of helping behavior on career satisfaction.

Fourth, the narrow sample scope limits the external validity of the research results. Our samples were selected from the construction industry in Beijing, China. Employees have different work characteristics, which can result in different types of helping behavior, and this can lead to different outcomes. Furthermore, China has a typical collectivist culture, which is different from most Western countries, such as the UK, the USA, and Australia. Prior studies have suggested that cultural differences shape the outcomes of helping behavior. Future research could re-examine the conceptual model in the context of working in different industries and cultures to further enhance the external validity of this study.

Fifth, due to the limitations of China’s epidemic prevention and control policies, the research team were not allowed access to the company; thus, we did not collect the data in person. The distribution and recovery of the questionnaires were mainly handled by human resource managers, which may have increased participants’ concern about privacy issues despite the training we provided for HR managers on the data collection process and precautions. Meanwhile, we also recorded a video explaining informed consent for HR managers to play for participants on the day of data collection and emphasized the protection of participants’ privacy and their right to quit the research at any time. However, due to the participation of HR managers in the process of data collection, the participants may still have worried that the questionnaire would be viewed by the managers. Researchers are encouraged to collect data personally after the end of the epidemic restrictions, and some variables can be evaluated by leaders or colleagues instead of by self-report to avoid such limitations.

## 6. Conclusions

This study deployed a two-wave survey and collected data from 203 full-time Chinese workers. The study examined the sequential mediating effects of perceived task demands and job strain on the relationship between helping behavior and career satisfaction. This study found that this indirect relationship depended on the level of the helpers’ strengths use. The indirect depleting impact of helping behavior on career satisfaction only emerged when strengths use was low. This study highlights the “dark side” of helping behavior and develops suggestions for practitioners to inhibit the negative influence of helping behavior on career satisfaction.

## Figures and Tables

**Table 1:** Results of confirmatory factor analysis.

Model	Factors	$\chi^{2}$	df	$\chi^{2}/\mathrm{df}$	$\Delta \chi^{2}$	CFI	TLI	RMSEA	SRMR
Five-Factor Model	EH, PT, JS, SU, CS	236.52	124	1.91		0.92	0.91	0.07	0.07
Four-Factor Model	EH + PT, JS, SU, CS	336.52	128	2.63	100.00 **	0.86	0.83	0.09	0.09
Three-Factor Model	EH + PT + JS, SU, CS	527.08	131	4.02	290.56 **	0.73	0.69	0.12	0.12
Two-Factor Model	EH + PT + JS + SU, CS	588.19	133	4.42	351.67 **	0.70	0.65	0.13	0.12
One-Factor Model	EH + PT + JS + SU + CS	721.00	134	5.38	484.48 **	0.61	0.55	0.15	0.13

Note: N = 203; ** *p* < 0.01. EH = helping behavior; PT = perceived task demands; JS = job strain; SU = strengths use; CS = career satisfaction.

**Table 2:** Means, standard deviations, and correlations.

	Mean	SD	1	2	3	4	5	6	7	8
1. Gender										
2. Age	32.27	6.34	0.05							
3. Education			0.20 **	0.07						
4. Career Satisfaction	3.81	0.62	−0.08	−0.01	0.03	(0.81)				
5. Helping Behavior	3.91	0.60	0.01	0.08	−0.05	0.36 **	(0.79)			
6. Perceived Task Demands	3.56	0.58	−0.10	0.13	0.05	0.22 **	0.28 **	(0.71)		
7. Job Strain	2.67	0.89	−0.03	0.17 *	0.02	−0.36 **	−0.07	0.25 **	(0.86)	
8. Strengths Use	3.73	0.61	−0.06	0.09	0.00	0.43 **	0.54 **	0.33 **	−0.12	(0.88)

Note: N = 203; * *p* < 0.05, ** *p* < 0.01. Values in parentheses are Cronbach’s alpha. For gender, 1 indicates male and 2 indicates female; for education, 1 indicates college degree or below, 2 indicates bachelor’s degree, and 3 indicates master’s degree or above.

**Table 3:** Results of bootstrapping analysis.

Paths	Effect	SE	95% CI	
			95% LL	95% LL
Mediating Effect				
Helping Behavior–Perceived Task Demands–Career Satisfaction	0.07	0.02	0.03	0.12
Helping Behavior–Job Strain–Career Satisfaction	0.01	0.04	−0.06	0.09
Multiple Mediation Effect	−0.04	0.01	−0.07	−0.01
Moderating Role of Strengths Use				
Low Strengths Use (M − SD)	0.79	0.14	0.51	1.07
High Strengths Use (M + SD)	0.18	0.14	−0.10	0.46
Difference	−0.61	0.18	−0.97	−0.25
Moderated Mediating Effect				
Low Strengths Use (M − SD)	−0.06	0.02	−0.11	−0.03
High Strengths Use (M + SD)	−0.01	0.01	−0.04	0.01
Difference	0.05	0.02	0.02	0.10

Note: Bootstrapping = 20,000 trials.

## Data Availability

The datasets that support the results of this paper are available from the corresponding author upon reasonable request.

## Supplementary Materials

The following supporting information can be downloaded at: https://www.mdpi.com/article/10.3390/ijerph20010161/s1, Survey items.
