# Supplementary material for: The Depleting Impact of Helping Behavior on Career Satisfaction: The Buffering Role of Strengths Use
Source: Int J Environ Res Public Health. 2022 Dec 22;20(1):161. doi: 10.3390/ijerph20010161 (PMC9819194; doi:10.3390/ijerph20010161)
Supplement: Supplementary file 1 [file ijerph-20-00161-s001.zip › ijerph-2050537-supplementary.pdf]

Survey items

1. Helping Behavior (Time 1, Yue et al., 2017)

HB1. I help other employees when it is clear their workload is too high.

HB2. I lend a helping hand to coworkers when needed.

HB3. I willingly assist other employees in meeting their job requirements.

2. Perceived Task Demands (Time 1; Williams & Alliger, 1994)

Measured the extent to which you are

PT1.rushed or hurried to complete their current activity.

PT2.challenged by the activity.

PT3.working hard on the activity.

PT4.putting forth high effort on the activity.

3. Strengths Use (Time 1; Woerkom, Oerlemans & Bakker, 2016)

SU1: I have Benefited in my work from my strengths.

SU2: I have conducted tasks that suit my strengths well.

SU3: I use my talents at work.

SU4: I have applied my personal qualities in my job.

4. Job Strain (Time 2; Schmitt, Hartog, & Belschak, 2016)

JS1: I have a lot of pressure at work.

JS2: I often feel to tense due to my work.

5. Career Satisfaction (Time 2; Eby & Butts, 2003)

CS1: Managers indicated the extent to which they agreed or disagreed.

CS2: I am satisfied with the progress I have made toward meeting my overall career goals.

CS3: I am satisfied with the progress I have made toward meeting my goals for income.

CS4: I am satisfied with the progress I have made toward meeting my goals for advancement.

CS5: I am satisfied with the progress I have made toward meeting my goals for the development of new skills.
